# No phenotypic resistance observed for most group-3 and -4 variants in *Mycobacterium tuberculosis* genes related to bedaquiline, clofazimine, delamanid, and pretomanid in a Central and West African context

**DOI:** 10.1128/spectrum.02005-26

**Published:** 2026-07-28

**Authors:** Faridath Massou, Klaas Dewaele, Anderson Gnamy, Ramata Balde, Wim Mulders, Conor J. Mehan, Bouke C. de Jong, Dissou Affolabi, Oren Tzfadia, Leen Rigouts

**Affiliations:** 1 Supranational Reference Laboratory of Tuberculosis, Cotonou, Bénin; 2 Mycobacteriology unit, Department of Biomedical Sciences, Institute of Tropical Medicine 37463 , Antwerp, Belgium; 3 Faculty of Pharmaceutical, Biomedical and Veterinary Sciences, University of Antwerp 26660, Antwerp, Belgium; 4 Centre for Systems Health and Integrated Metabolic Research, Nottingham Trent University 6122, Nottingham, United Kingdom; Pontificia Universidad Catolica de Chile, Santiago, Chile

**Keywords:** minimum inhibitory concentration, whole-genome sequencing, *Mycobacterium tuberculosis*

## Abstract

The interpretation of genetic variants’ association (or not) with phenotypic resistance to newly introduced and repurposed antituberculosis drugs remains challenging, as many mutations detected by whole-genome sequencing (WGS) are classified as of uncertain significance (group 3) or not associated with resistance-interim (group 4) by the World Health Organization (WHO) mutation catalog v2. We evaluated the phenotypic impact of such variants on minimum inhibitory concentrations (MICs) for bedaquiline (BDQ), clofazimine (CFZ), delamanid (DLM), and pretomanid (PA) in *Mycobacterium tuberculosis* complex isolates from the multi-country DIAMA cohort in sub-Saharan Africa (SSA), which recruited RR/RS-TB patients naïve to these drugs. Among 1,475 isolates with available WGS data, 163 variants met eligibility criteria; due to viable strain unavailability, 89 isolates carrying 29 unique BDQ/CFZ-related and 60 unique DLM/PA-related variants were tested for MIC determination using broth microdilution. Additional structural modeling was performed to explore potential effects of amino-acid substitutions on protein stability. Among BDQ/CFZ-related variants, MICs above the critical concentrations (CCs) were consistently associated with *mmpR5* variants, whereas variants in *atpE*, *pepQ*, and *Rv1979c* were not. DLM/PA variants (*ddn, fbiA–D,* and *fgd1*) were frequently detected as non-fixed populations, yet rarely yielding MIC values above the CC. Predicted structural destabilization showed no consistent association with MIC values or variant fixation status. Under the conditions tested, phenotypic resistance was not detected for most group 3 and 4 variants detected by WGS. Our data provide evidence from SSA to support improved interpretation of resistance-associated mutations for new and repurposed antituberculosis drugs.

**IMPORTANCE:**

Whole-genome sequencing increasingly detects *Mycobacterium tuberculosis complex* mutations classified by the World Health Organization (WHO) mutation catalog v2 as group 3 variants of uncertain significance or group 4 variants not associated with resistance-interim, limiting reliable prediction of resistance to new and repurposed antituberculosis drugs. By generating minimum inhibitory concentration (MIC) data for such variants identified in a multi-country sub-Saharan African cohort, this study provides phenotypic evidence to support future refinement and expansion of the WHO mutation catalog v2. Notably, *mmpR5* variants associated with elevated bedaquiline/clofazimine MICs were identified in eight isolates, suggesting that some patients in this cohort may have harbored pre-existing resistance-associated variants yet remained potentially eligible for bedaquiline-containing regimens. These findings contribute to improving the interpretation of genomic resistance data and strengthening surveillance of resistance to bedaquiline, clofazimine, delamanid, and pretomanid.

## **I**NTRODUCTION

In recent years, treatment options for multidrug-resistant tuberculosis (MDR-TB) have been broadened through the introduction of novel agents, including bedaquiline (BDQ), delamanid (DLM), and pretomanid (PA), as well as the repurposing of clofazimine (CFZ), originally developed for leprosy treatment (1). BDQ, a diarylquinoline, targets the mycobacterial ATP synthase, while DLM and PA are nitroimidazoles that inhibit mycolic acid biosynthesis and cause respiratory poisoning (2). CFZ, a riminophenazine, exerts antimycobacterial activity through membrane destabilization and generation of reactive oxygen species (2). Together, these agents are endorsed by the World Health Organization (WHO) as components of MDR-TB regimens (1).

However, resistance mechanisms to these newer drugs are complex and involve multiple genetic determinants, including mutations in both essential and non-essential genes, regulatory genes, and efflux pump systems (3). These mechanisms display substantial genetic diversity and have even been reported in treatment-naïve isolates (4), highlighting the importance of comprehensive phenotypic and genotypic characterization of identified variants.

To support the association (or not) of observed genetic variants with phenotypic resistance, the WHO has published a catalog of *Mycobacterium tuberculosis* complex (MTBC) mutations (3). Genetic variants are classified according to the strength of evidence linking them to resistance, including five categories: associated with resistance (group 1), associated with resistance-interim (group 2), uncertain significance (group 3), not associated with resistance-interim (group 4), and not associated with resistance (group 5). In addition, the increasing use of whole-genome sequencing (WGS) in TB research continues to reveal novel variants, many of which are either group 3 or group 4. These variants limit the predictive power of WGS for resistance detection, necessitating the introduction of additional grading rules for interpreting their association with drug resistance (3).

The available evidence underlying the WHO catalog is largely derived from cohorts in Asia, Eastern Europe, and South America. Sub-Saharan Africa (SSA), despite bearing a notable share of MDR/RR-TB, is comparatively underrepresented, with the exception of South Africa (3). As a result, the catalog may not yet fully capture the spectrum of resistance-associated variants circulating globally.

In this study, we investigated the effect of group-3 and -4 mutations on the minimum inhibitory concentrations (MICs) of BDQ, DLM, CFZ, and PA, by combining WGS-based data with broth microdilution (BMD) testing.

## RESULTS

### Characteristics of the study isolates

**Fig 1:**
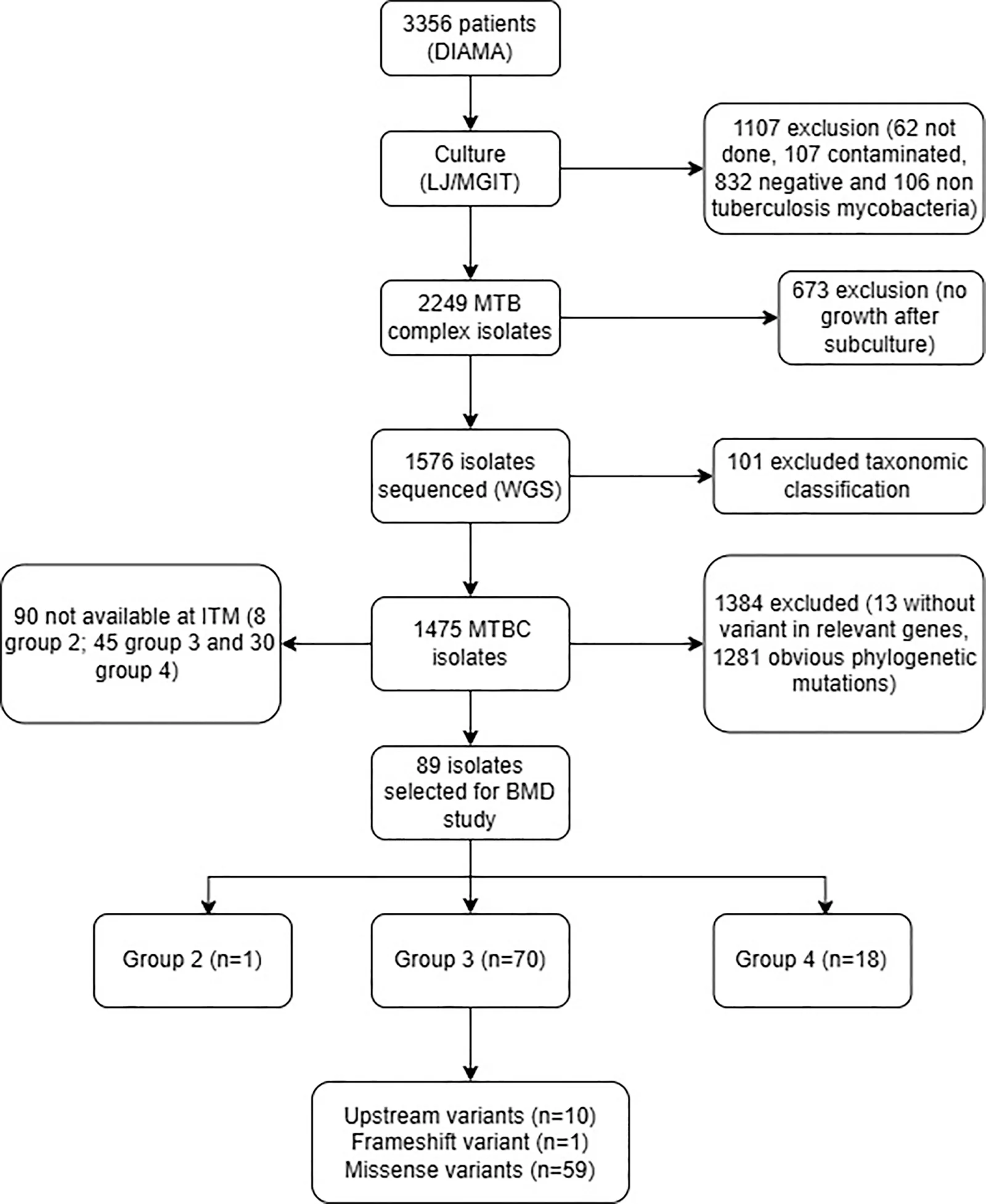
Flowchart of sample selection from the DIAMA study for inclusion in the BMD analysis. A total of 3,356 patients were included in the DIAMA study. After culture (LJ/MGIT), 2,249 MTBC isolates were obtained. Following exclusions (contamination, negative cultures, non-tuberculous mycobacteria, or lack of growth after subculture), 1,576 isolates were successfully sequenced by WGS. After quality control and taxonomic classification, 1,475 MTBC isolates remained. Additional exclusions based on the absence of relevant resistance mutations, presence of phylogenetic variants, or sample unavailability at the ITM resulted in a final selection of 89 isolates included in the BMD study. Variants are grouped according to the WHO mutation catalog v2 classification group definitions (group 2: associated with resistance interim; group 3: uncertain significance; group 4: not associated with resistance interim) and by variant type (upstream, frameshift, and missense variants). BMD, broth microdilution; ITM, Institute of Tropical Medicine; LJ, Löwenstein-Jensen; MGIT, mycobacterial growth indicator tube; MTBC, *Mycobacterium tuberculosis* complex; WGS, whole-genome sequencing.

Among 1,475 MTBC isolates with valid WGS data (Fig. 1), 179 (12.1%) carried 248 mutations in genes associated with BDQ, CFZ, DLM, or PA resistance spanning WHO catalog groups 2 (*n* = 14), 3 (*n* = 155), and 4 (*n* = 48). Of these, 163 variants (group 3: *n* = 115, group 4: *n* = 48) met the eligibility criteria defined for this study. Due to non-availability of viable strains at the Institute of Tropical Medicine (ITM), 89 isolates were available for MIC testing, comprising 29 unique BDQ-/CFZ-related variants and 64 unique DLM-/PA-related variants (File S1): 18 synonymous variants (group 4), 59 missense variants (group 3), 10 upstream variants (group 3), one frameshift variant (group 3), and one stop-gained variant (group 2). The latter was unintentionally included based on initial classification but was retained for analytical transparency. One-third of these isolates (33/89, 37.1%) were classified as soft fail using TBProfiler v6.6.6 (QC details provided in File S1).

Included isolates originated from seven countries (File S2, Table 1), predominantly from Cameroon (42.9%), and were mainly lineage 4 (72/89; 82.02%), followed by lineage 5 (*n* = 6), lineage 3 (*n* = 5), lineage 2 (*n* = 4), and single isolates of lineage 6 and lineage 8. Rifampicin resistance was observed in 56.2% (50/89) of isolates, with most originating from retreatment cases (70/89; 78.7%), while fluoroquinolone (FQ) resistance was rare (2/89; 2.2%).

### Analysis of mutations for BDQ/CFZ resistance

**Fig 2:**
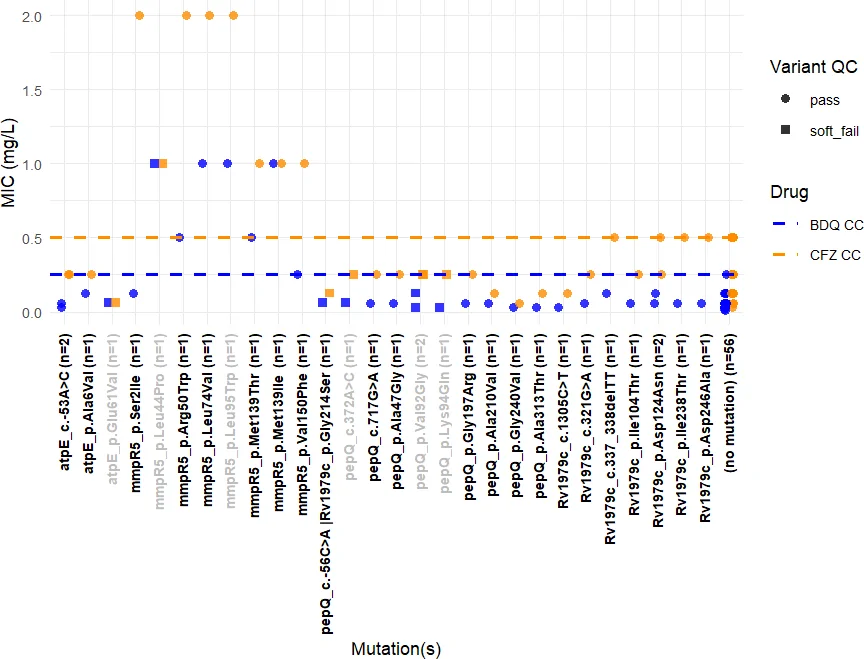
Individual MIC values for BDQ and CFZ per variant tested. Scatter plot displaying individual MIC values for BDQ and CFZ across mutations. The *X*-axis indicates mutations according to their genomic positions, and the *Y*-axis indicates the corresponding MIC values (mg/L). Each point represents a single isolate, with blue points corresponding to BDQ and orange points to CFZ. Dashed horizontal lines indicate the critical concentration thresholds. Mutations displayed in gray on the *X*-axis correspond to non-fixed variants (frequency <75%), whereas mutations shown in black represent fixed variants. Point shape indicates the variant quality control status according to TB-Profiler (v6.6): dots represent PASS variants (sequencing depth ≥10 reads, allele frequency ≥10%, and ≥3 reads supporting the alternate allele on both forward and reverse strands), whereas squares represent SOFT_FAIL variants (variants for which one or more of these criteria are not met). In this data set, all variants had an allele frequency of ≥10%.

In all, 31 isolates carried eligible mutations in genes potentially associated with resistance to BDQ/CFZ, representing 29 distinct variants: *mmpR5* (*n* = 8), *atpE* (*n* = 3), *pepQ* (*n* = 10), and *Rv1979c* (*n* = 8). Over half of the isolates (17/31; 59.0%) were rifampicin-resistant. Most variants (24/29, 82.8%) passed TB-Profiler QC filters (Fig. 2), and valid MIC results were obtained for all isolates. Among 57 wild-type isolates (for BDQ/CFZ-associated genes), 13 showed CFZ MICs at the CC, consistent with CFZ phenotypic variability (5), while only one reached the BDQ-CC; this isolate carried an *fbiC* p.Arg619Cys variant and had elevated DLM MIC. As expected, MICs remained at or below the CC (Fig. 2) for most group 3 and 4 variants, for both BDQ (*n* = 23/29) and CFZ (*n* = 21/29).

**Fig 3:**
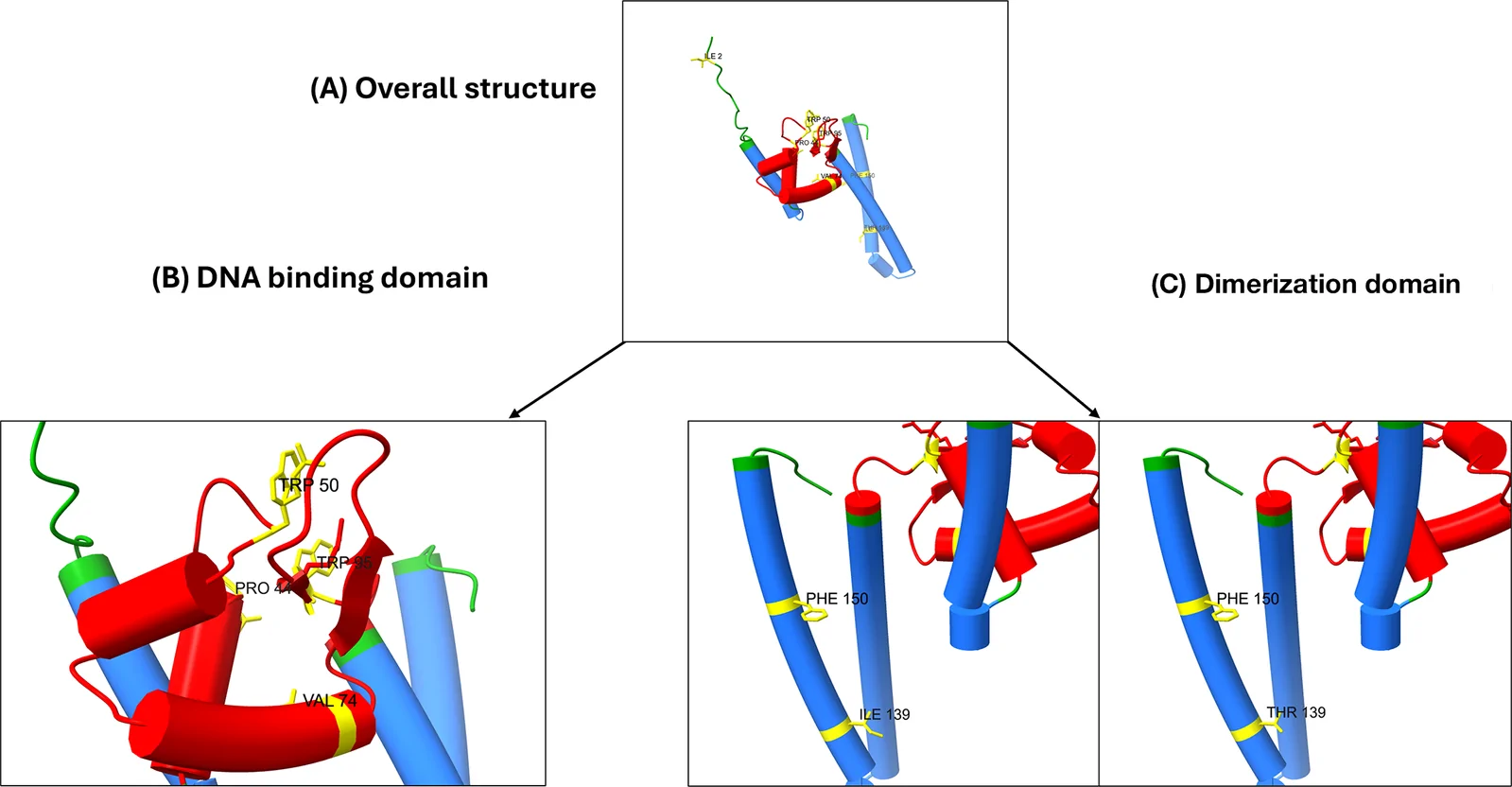
Predicted three-dimensional structure of mmpR5. (**A**) Overall AlphaFold-predicted structure of mmpR5 shown in cartoon representation. The DNA-binding domain is shown in red, the dimerization domains in blue, and flexible terminal regions in green. Amino-acid substitutions associated with elevated minimum inhibitory concentrations are highlighted in yellow. (**B**) Enlarged view of the DNA-binding domain highlighting resistance-associated substitutions. (**C**) Enlarged view of the dimerization domain showing mutations located within the helical interface

Four isolates carrying three different *atpE* variants were tested: two harbored a fixed promoter variant (c.-53A > C, located outside the WHO-defined upstream region; File S1), while two carried intragenic variants, including a fixed p.Ala6Val substitution and a minority (12%) soft-failed p.Glu61Val variant (Fig. 2). None of these isolates presented an MIC above the CC. Mapping of the two intragenic variants onto the predicted ATP-synthase F_0_ structure showed that the substitutions were located within the transmembrane helices of the c-ring without clustering in known functional hotspots (Fig. 3).

Eight isolates carrying eight distinct *mmpR5* coding-region variants (six fixed and two non-fixed) were tested. All isolates exhibited CFZ-MIC values exceeding the CC, of which six also displayed an MIC above the CC for BDQ. Variants were located either in the DNA-binding domain (codons 34–99: Leu44Pro, Arg50Trp, Leu74Val, and Leu95Trp), the dimerization domain (codons 16–32 and 101–160: Val150Phe, Met139Ile, and Met139Thr) or the N-terminal region (Ser2Ile). All eight isolates were observed in distinct lineage backgrounds, were predominantly retreatment cases, and were all FQ susceptible (Table 1; File S2). Structural mapping onto the AlphaFold-predicted *mmpR5* structure confirmed that these eight substitutions were distributed across the functional domains involved in transcriptional regulation and protein–protein interactions (Fig. 3). Two *mmpR5* variants did not surpass the CC for BDQ: one isolate with an MIC of 0.125 mg/L carried a fixed *mmpR5* Ser2Ala variant along a fixed *mmpL5* Leu836Phe variant (group 3), while the second carried a fixed *mmpR5* Val150Phe variant and had an MIC at the CC (0.25 mg/L).

**TABLE 1:** Detailed characteristics of isolates with MIC above the CC for ≥ 1 drug^*a*^^,^^*b*^

ID	Sub lineage	Country	TB history	*rpoB* mutation(s)	*gyrA/B* mutation	*mmpR5* mutation	WHO Cat.	*fbi/fgd* pathway mutation	BDQ MIC	CFZ MIC	DLM MIC	PA MIC	FoldX* (Kcal/mol)	Dynamut2* (Kcal/mol)
CT202000810	L4.1	Guinea	Relapse	–	–	**Ser2Ile**	3	NA	0.125	**1**	0.008	0.06	0.44	−0.63
CT201903751	L6.3	Guinea	Relapse	Ser450Leu	–	**Leu44Pro**	3	*fgd1*_p.Lys296Glu	**1**	**1**	≤0.002	≤0.008	3.77	−0.71
CT201903922	L4.1.3	Guinea	New case	Leu452Pro	–	**Arg50Trp**	3	*fbiC*_c.1680c > t	**0.5**	**2**	0.008	0.125	0.49	−0.07
CT202001446	L2.2.1	Guinea	New case	Ser450Gln	–	**Leu74Val**	3	NA	**1**	**2**	0.008	0.25	3.50	−2.29
CT201903769	L2.2.1	Guinea	New case	His445Ala + Val170Phe	–	**Leu95Trp**	3	NA	**1**	**2**	0.008	0.25	3.55	−2.40
CT201901149	L5.2	Cameroon	Relapse	His445Ala	–	**Met139Ile**	3	*fbiA*_p.Ile223Val	**1**	**1**	0.008	0.125	1.05	−0.46
CT202003015	L3	Ethiopia	Failure	Leu452Pro	–	**Met139Thr**	3	NA	**0.5**	**1**	0.008	0.125	1.85	−0.88
CT201901240	L3	Cameroon	Relapse	–		**Val150Phe**	3		**0.25**	**1**	0.015	0.25	−0.06	−1.81
CT202001451	L4.8	Guinea	New case	Ser450Leu	–	NA	3	***fbiC*_p.Arg619Cys**	0.25	0.06	**0.25**	0.125	0.90	0.5

^*a*^ L, lineage; RIF, rifampicin; FQ, fluoroquinolones; CC, critical concentration; MIC, minimum inhibitory concentration; BDQ, bedaquiline; CFZ, clofazimine; DLM, delamanid; PA, pretomanid; –, wild type; NA, absence of potential mutations associated with high MIC. WHO Cat, classification according to the WHO catalog v2.

^*b*^ Bold indicates MIC values above the critical concentration for the corresponding drug, the associated mutation, and/or ΔΔG values with a predicted destabilizing effect on protein stability (FoldX) or folding dynamics (DynaMut2), FoldX < –0.5 kcal/mol indicates stabilization, –0.5 to +0.5 neutral effects, and > +0.5 destabilization. For DynaMut2, –0.5 to +0.5 indicates neutral effects, < –0.5 destabilization, and > +0.5 stabilization. Destabilizing values are shown in bold. *Values displayed represent the energy for the gene/mutation depicted in bold.

Among the 11 isolates carrying 10 distinct *pepQ* variants, two were synonymous mutations (c.372A > C and c.717G > A), one of which was not fixed, and one isolate harbored a fixed promoter variant (c.-56C > A located outside the WHO-defined upstream region; File S1). The remaining isolates carried seven different amino-acid substitutions (Ala47Gly, Val92Gly, Lys94Gln, Gly197Arg, Ala210Val, Gly240Val, and Ala313Thr), of which five were fixed. None of the *pepQ* variants was associated with reduced phenotypic susceptibility to BDQ or CFZ.

Nine isolates carried eight distinct fixed *Rv1979c* variants. Three were coding-region variants, including one frameshift mutation (c.337-338delTT) and two synonymous mutations (c.321G > A and c.1305C > T), while five isolates carried amino-acid substitutions (Ile104Thr, Asp124Asn, Gly214Ser, Ile238Thr, and Asp246Ala). None of these nine isolates exhibited MIC values above the CC for BDQ or CFZ; however, variants c.337-338delTT, Gly214Ser, Ile238Thr, and Asp246Ala displayed CFZ-MIC values at the CC, a finding that should be interpreted in light of the known CFZ phenotypic variability (5).

### Analysis of mutations for DLM/PA resistance

**Fig 4:**
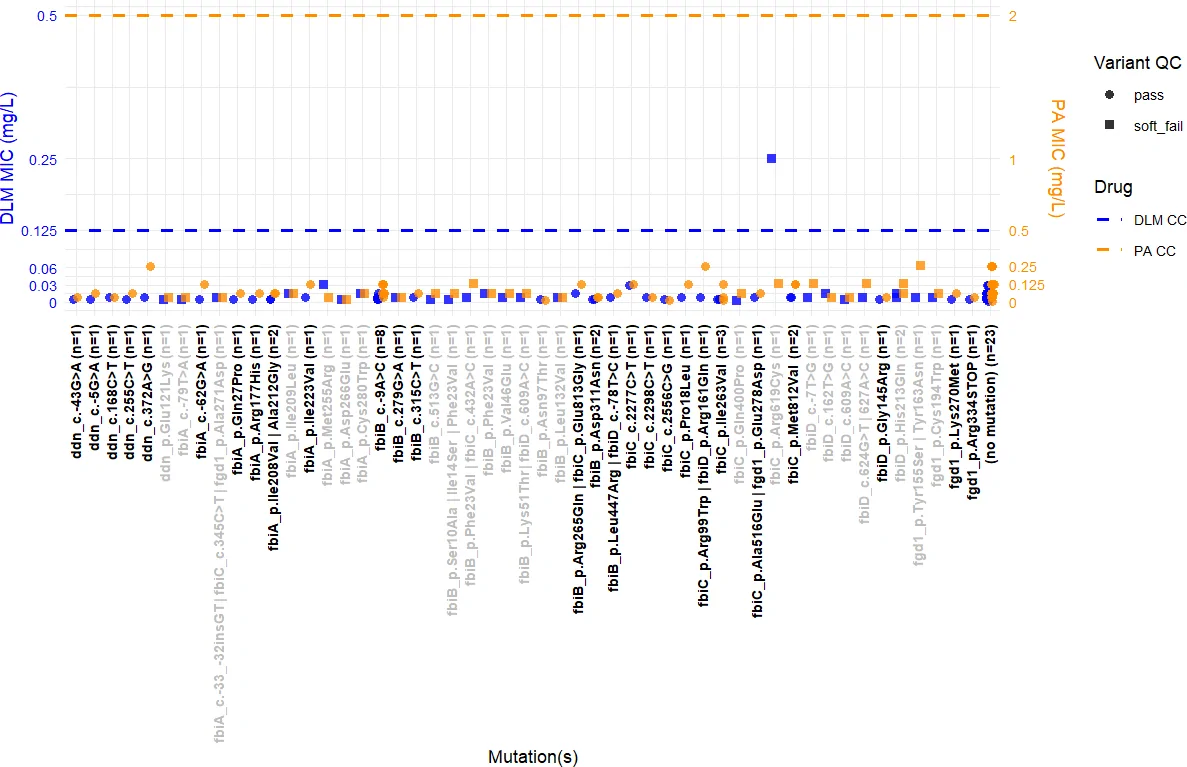
Individual MIC values for DLM and PA per variant tested. Scatter plot displaying individual MIC values for DLM and PA across mutations. The *x*-axis indicates mutations according to their genomic positions. The left *y*-axis indicates DLM MIC values (mg/L), and the right *y*-axis indicates PA MIC values (mg/L), with independent scales reflecting the different critical concentrations for each drug. Each point represents a single isolate, with blue points corresponding to DLM and orange points to PA. Dashed horizontal lines indicate the critical concentration thresholds. Mutations displayed in gray on the *x*-axis correspond to non-fixed variants (frequency <75%), whereas mutations shown in black represent fixed variants. Point shape indicates the variant quality control status according to TB-Profiler (v6.6): dots represent PASS variants (sequencing depth ≥10 reads, allele frequency ≥10%, and ≥3 reads supporting the alternate allele on both forward and reverse strands), whereas squares represent SOFT_FAIL variants (variants for which one or more of these criteria are not met). In this data set, all variants had an allele frequency of ≥10%.

**Fig 5:**
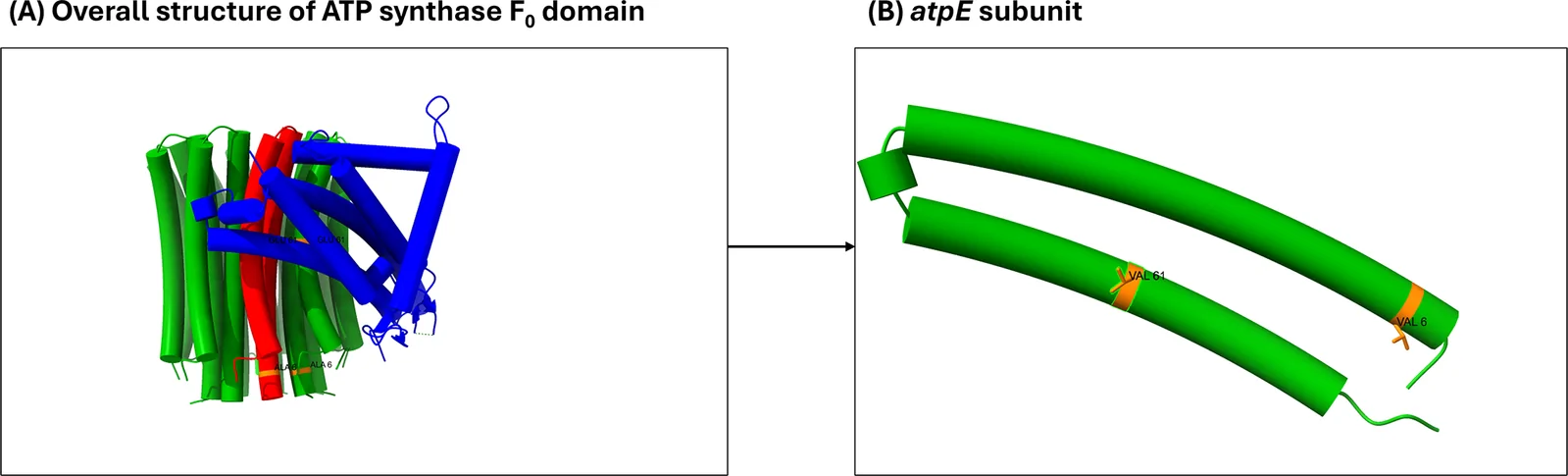
Predicted three-dimensional structure of atpE within the ATP synthase F₀ domain. (**A**) Overall predicted structure of the mycobacterial ATP synthase F₀ with the c-ring (atpE ×9, green) and the a subunit (atpB ×1, blue) are shown. One atpE subunit is highlighted in red. (**B**) Enlarged view of the atpE subunit highlighting in orange susceptible-associated amino-acid substitutions.

Sixty-four isolates carried 60 unique variants in genes potentially associated with resistance to DLM/PA: *ddn* (*n* = 6), *fbiA* (*n* = 12), *fbiB* (*n* = 13), *fbiC* (*n* = 13), *fbiD* (Rv2983; *n* = 9), and *fgd1* (*n* = 7). Half of these variants (30/60; 50.0%) appeared as non-fixed and 28 (46.7%) did not pass the TB-Profiler QC filter predominantly because reads supporting the alternate allele were detected on only one DNA strand, failing the criterion of ≥3 supporting reads on both DNA strands (Fig. 4; File S1). Valid MIC results were obtained for all isolates. None of the wild-type isolates (for DLM/PA-associated genes) reached the CC for either drug, and most isolates exhibited MICs below the CC for DLM (*n* = 63) and PA (*n* = 64) (Fig. 5).

Six isolates carrying six distinct *ddn* variants were tested, including two fixed promoter variants (c.-5G > A and c.-43G > A, both located within the WHO-defined upstream region; File S1) and four intragenic variants. Coding-region variants included three fixed synonymous substitutions (c.168C > T, c.255C > T, and c.372A > G) and one minority amino-acid substitution (p.Glu121Lys). All six isolates showed MIC values comparable to wild-type isolates.

In all, 54 isolates carried 47 distinct variants across genes involved in F420 cofactor biosynthesis (*fbiA*, *fbiB*, *fbiC*, and *fbiD*), with variable fixation profiles including 53.2% (25/47) non-fixed variants. These variants comprised six promoter mutations (including one located outside the WHO-defined upstream region; File S1), 11 synonymous mutations, and 30 amino-acid substitutions (Fig. 4). Among these, only the non-fixed *fbiC* Arg619Cys substitution yielded an MIC above the CC for DLM. This isolate belongs to lineage 4.8 and came from a patient never exposed to any TB treatment drugs (Table 1).

Six isolates carried seven *fgd1* variants, all located within the coding region. Five were amino-acid substitutions, including two fixed variants (Lys270Met and Glu278Asp), three were non-fixed variants (Tyr155Ser, Tyr163Asn, and Cys194Trp), and one isolate harbored a fixed premature stop codon (Arg334STOP). MIC values for all six isolates remained within the susceptible range.

### Structural analysis of resistance-associated variants

Predicted structural effects of amino-acid substitutions were assessed using FoldX and DynaMut2 (File S2, Tables 2 and 3). Across BDQ/CFZ and DLM/PA-associated genes, predicted stability effects varied widely across variants and genes, ranging from neutral to strongly destabilizing.

All *mmpR5* variants were associated with CFZ MICs above the CC, although predicted protein stability changes showed substantial variability. Conversely, several amino-acid substitutions across *atpE, pepQ*, *Rv1979c*, *ddn*, *fbiA*, *fbiB*, *fbiC*, *fbiD*, and *fgd1* genes were predicted to destabilize protein stability without corresponding significant increases in MIC values. Among DLM/PA-associated genes, MIC values above the CC were observed only for the *fbiC* Arg619Cys substitution despite heterogeneous predicted structural effects across variants.

No statistical correlation was observed between predicted structural effects and MIC values for BDQ/CFZ-related variants (Spearman *ρ* = 0.10, *P* = 0.52) or for DLM/PA-related variants (*ρ* = 0.02, *P* = 0.85), indicating the absence of a quantitative relationship between predicted structural effects and MIC values (File S3). Also, no significant difference in predicted structural effect was observed between fixed and non-fixed variants (*P* = 0.25), suggesting that the variant proportion likely did not impact protein stability.

Overall, these findings indicate that predicted structural destabilization alone does not reliably predict phenotypic resistance levels across BDQ/CFZ and DLM/PA resistance-associated genes.

## DISCUSSION

This study provides phenotypic evidence to support the interpretation of MTBC mutations in the context of the WHO mutation catalog v2, with a specific focus on newer and repurposed antituberculosis drugs, contributing data from under-represented Central- and West-African countries.

Consistent with their group-4 classification, none of the 18 synonymous variants were associated with MICs above the CC. Likewise, group-3 upstream variants including three outside genomic boundaries defined by the WHO mutation catalog v2 and the *Rv1979c* frameshift were not associated with MIC increases. Among the 59 missense variants (group 3), nine yielded an MIC above the CC for at least one drug. The only group-2 variant (*fgd1* p.Arg334STOP) did not have an MIC above the CCs. While loss-of-function mutations in *fgd1* are generally expected to abolish F420-dependent prodrug activation and confer DLM/PA resistance (3), this discordance may reflect an epistatic compensatory mechanism (isolate carries additional mutations in *ddn* and *fbiC*), residual enzymatic activity of the near-complete truncated protein (stop at codon 334/342), or limitations inherent to single time-point phenotypic testing.

Variants in *atpE*, encoding the BDQ target ATP synthase, were rare and not associated with elevated MICs in this data set. This observation is consistent with previous reports showing that *atpE* mutations occur infrequently in clinical MTBC isolates, likely because of the substantial fitness cost associated with alterations in this essential gene (6). Notably, one isolate carried a minority variant p.Glu61Val (12%, soft_fail), at a codon located in the proton translocation site of the ATP complex, presumed to impact BDQ activity. The WHO mutation catalog v2 classifies the p.Glu61Asp variant at that same codon as associated with interim resistance (3). Despite the expected functional impact of this disruptive substitution, no BDQ-MIC elevation was observed for our isolate, likely reflecting incomplete fixation of the variant, possible restoration of the wild-type population during subculture prior to MIC determination, or a sequencing artifact given the soft_fail status.

Elevated BDQ and CFZ MICs above the CCs were exclusively reported for *mmpR5* variants, supporting that efflux-mediated resistance remains the dominant mechanism for these drugs (7, 8). All such isolates displayed CFZ-MIC values above the CC, with almost all also showing BDQ cross-resistance, consistent with the shared efflux-mediated resistance pathway. Two exceptions were observed: one isolate (*mmpR5* Val150Phe) had a BDQ-MIC at the CC, and the second isolate (*mmpR5* Ser2Ile) had a BDQ-MIC just below the CC. Both should be interpreted with caution given the known variability near the CC, particularly for CFZ (9). For the latter, additional *mmpL5* variants were present; although not predicted to cause loss-of-function change, a potential epistatic interaction affecting efflux-pump functionality cannot be excluded (3).

The isolates with BDQ/CFZ-MICs above the CC were all identified in rifampicin-resistant yet FQ-susceptible backgrounds. Affected patients would have been eligible for current BDQ-containing regimens, including the 6-month BPaLM regimen or alternative BDQ-based combinations such as BDQ-DLM-linezolid-FQ-CFZ regimens (e.g., BDLLfxC-based regimens) for MDR/RR-TB treatment, had they been diagnosed today (1). Overall, the proportions of rifampicin and FQ resistance in our study were broadly consistent with reported epidemiological trends for this geographical region (10).

As low-complexity rapid molecular diagnostics are currently limited to FQ-resistance detection (Xpert MTB/XDR [Cepheid, USA] or GenotypMTBDRsl [Brucker, Germany]), resistance to BDQ or CFZ may remain undetected at treatment initiation. While targeted next-generation sequencing is endorsed by WHO for resistance detection (11), its infrastructure and expertise requirements limit its routine deployment in most high-burden settings. The large-scale programmatic blinded use of BDQ therefore raises concern that pre-existing resistance-associated variants, including minority *mmpR5* variants detected in two out of eight *mmpR5* variants in our data set could be unintentionally selected or amplified during treatment, highlighting the urgent need for rapid diagnostic tools capable of detecting BDQ/CFZ resistance prior to regimen initiation. Notably, all tested isolates were collected before the programmatic rollout of BDQ-containing regimens in the participating countries, supporting the hypothesis that efflux-mediated resistance-associated variants may pre-exist treatment initiation (12). However, incomplete treatment histories and potential prior exposure to CFZ through non-standardized regimens cannot be fully excluded.

Variants detected in *pepQ* and *Rv1979c,* both tier-1 genes for CFZ (3), were all associated with MICs at or below the CC for BDQ and CFZ. Of note, *Rv1979c* is classified as tier 2 for BDQ (3), further supporting its limited role as a primary resistance determinant. These findings support previous studies suggesting these genes may instead represent secondary or permissive adaptations whose phenotypic impact depends on genetic background or additional resistance mechanisms (5, 13).

A higher proportion of genetic variants was identified in genes involved in DLM/PA activation (*ddn, fbiA-D,* and *fgd1*) than in BDQ/CFZ-associated genes, with a higher proportion of non-fixed variants (50.0% vs 20.7% for BDQ/CFZ). These genes belong to the F420-dependent activation pathway and are generally considered non-essential for *in vitro* growth (14). Resistance frequently results from loss-of-function mutations that abolish DLM/PA-prodrug activation without compromising bacterial viability (13, 14). The large mutational target size of this pathway likely facilitates the early emergence of non-fixed subpopulations, although these variants were not consistently associated with elevated MICs in our data set.

The predominance of low MIC values despite numerous detected variants suggests that many mutations in the F420 pathway may represent early evolutionary events or natural polymorphisms without immediate phenotypic impact *in vitro* (15). However, their clinical significance remains uncertain, particularly under drug pressure, where selected subpopulations or mutations affecting prodrug activation could potentially contribute to treatment failure or facilitate the emergence of higher-level resistance (3, 16). Population-level exposure to other nitroimidazole compounds, such as metronidazole, which is widely used empirically for gastrointestinal, gynecological, and anaerobic infections in many high-burden settings (17), may also contribute to the selection or maintenance of pre-existing minor variants affecting related reductive activation pathways, although direct epidemiological evidence remains limited (18). Isolate CT202001451 illustrates this complexity, despite no documented prior exposure to nitroimidazoles. It carried *fbiC* p.Arg619Cys (12%, soft_fail) alongside a co-occurring *ddn* c.-115T > A variant (15%), the latter located outside both the WHO catalogue-defined upstream boundary and our inclusion criteria. This isolate also has a BDQ-MIC at the CC without carrying any variant in known genes associated with BDQ/CFZ resistance. Although causality cannot be inferred, this observation is compatible with emerging *in vitro* evidence showing that CFZ exposure can select for *fbiC* mutations associated with cross-resistance to nitroimidazoles, typically after acquisition of *mmpR5/Rv0678* mutations (19).

Unlike BDQ/CFZ, resistance to nitroimidazoles involves multiple genes of the F420 activation pathway, complicating diagnostic development (20). Therefore, although surveillance for all four drugs remains essential, the immediate diagnostic priority may lie in improving detection of BDQ/CFZ resistance. Nevertheless, the multigenic and heterogeneous resistance mechanisms affecting DLM and PA underscore the longer-term challenge of developing comprehensive molecular diagnostics capable of reliably capturing clinically all relevant resistance determinants.

Structural modeling revealed variability in predicted stability effects without consistent association with MIC elevation or heteroresistance status, consistent with previous studies (15, 20) combining structural predictions and phenotypic data for proteins of interest including *atpE*, *ddn*, *fgd1,* and *mmpR5*. Although protein destabilization has been reported as a contributor to antimicrobial resistance in non-mycobacterial species (21), stability-based *in silico* predictions did not prove informative in our data set to reliably predict resistance phenotypes in MTBC.

Finally, the diversity of variants observed in the genes of interest likely reflects underlying genetic diversity within our MTBC population rather than drug-specific selective pressure, particularly given the absence of documented prior exposure to these drugs and the multi-country nature of our study. This interpretation is consistent with the observations from South Africa, where reported predominance of lineage-2 isolates among baseline BDQ-resistant cases (22) most likely mirrors the local lineage distribution. Whether phylogenetic background genuinely shapes the emergence of resistance to newer anti-TB drugs, as has been established for *rpoB* mutations (23), remains to be determined as larger data sets accumulate.

This study has some limitations. Most mutations were represented by single isolates, limiting firm variant-level conclusions. For isolates carrying co-occurring mutations either within the same gene or across multiple genes, predominantly among DLM/PA-related variants, epistatic or additive interactions cannot be excluded, further limiting variant-level conclusions for this specific subset. MIC interpretation relied on literature-derived CCs for BDQ, CFZ, and DLM, as standardized WHO CC and clinical breakpoint values are not established yet, which may have introduced uncertainty, particularly for isolates with borderline MIC values. Also, structural predictions were based on monomeric protein models reflecting changes in folding stability and did not account for cofactor binding or catalytic activity known in F420-pathway enzymes whose function depends on interactions with F420 and substrate molecules. The absence of correlation between predicted destabilization and MIC values should therefore be interpreted within these computational constraints. Furthermore, as WGS and MIC determination were performed on independent subcultures from the same frozen stock, minority variants may have been lost or enriched during subculture, and the absence of MIC elevation in such isolates should be interpreted with caution. Finally, some variants were classified as soft fail under current TB-Profiler quality control criteria, mainly due to DNA strand imbalance in the context of low sequencing depth. Whether this reflects sequencing artifacts or true low-frequency variants cannot be determined from the available data. Although retained for completeness, these variants should be interpreted with caution, as they may represent low-confidence calls and affect genotype-phenotype associations. Nevertheless, one of the eight BDQ/CFZ isolates with MICs above the CC and the single DLM/PA-resistant isolate were soft fails. Isolates from Cameroon were overrepresented, reflecting operational factors. In addition, nearly half of the eligible isolates could not be phenotypically tested due to strain unavailability, potentially affecting the observed distribution. Finally, strain selection partly relied on excluding highly prevalent lineage-associated variants, yet these may still confer intrinsic resistance, which requires further testing.

Overall, these findings highlight the complexity of interpreting resistance-associated variants to new and repurposed antituberculosis drugs and emphasize the need to combine genomic, phenotypic, and clinical data to support reliable resistance prediction and treatment decision-making.

## MATERIALS AND METHODS

### Study design

This study was nested within the EDCTP2-funded DIAMA diagnostic trial (DRIA2014-326), which enrolled approximately 3,356 TB patients across nine SSA countries (Benin, Cameroon, Democratic Republic of Congo, Ethiopia, Guinea, Mali, Nigeria, Rwanda, and Senegal) between 2017 and 2021. The study included rifampicin-resistant isolates from new (naïve for TB treatment) and retreatment cases, complemented with an equal number of rifampicin-susceptible isolates from retreatment cases to increase sample size and because, despite remaining susceptible to rifampicin, their history of prior drug exposure confers a higher probability of harboring mutations in genes beyond *rpoB* compared to new cases. Retreatment cases were defined as treatment failure, return after default, or relapse of first-line TB treatment. At the time of sample collection, none of the drugs tested were used to treat any of the included patients. Among all patients included in the DIAMA study, 1,465 isolates had high-quality WGS and metadata available, including information on country of origin, TB treatment history, rifampicin resistance, phylogenetic (sub-) lineage, and mutations in genes of interest (Fig. 1).

### WGS analysis

WGS was performed on MTBC isolates using the Illumina Novaseq 6000 platform (Illumina, San Diego, CA, USA), with a mean coverage of 30×. Raw sequencing reads were first subjected to taxonomic classification using Centrifuge v1.0.4 (24), and reads not assigned to MTBC were removed prior to downstream analysis to reduce the risk of misalignment artifacts and false variant calls.

Filtered reads were then analyzed with TB-Profiler v6.6.6 (25), which aligns reads to the MTB H37Rv reference genome (GenBank accession NC_000962.3), calls single-nucleotide polymorphisms (SNPs) and small insertions/deletions in drug-resistance-associated genes, and assigns MTBC lineages. TB-Profiler also provides resistance predictions based on its curated database. Variants with an allele frequency <10% were excluded by TB-Profiler v6.6.6 and therefore not considered true variants in this study. For this analysis, variants were further classified based on allele frequency, with variants between 10% and 75% considered non-fixed and variants ≥75% considered fixed, in line with the allele frequency threshold used in the WHO mutation catalog v2 (3). Variants were also classified with their quality control (QC) status as defined by TB-Profiler 6.6.6 into the categories “pass” (sequencing depth ≥10 reads, allele frequency ≥10% and ≥3 reads supporting the alternate allele on both forward and reverse strands) and “soft fail” (variants not meeting one or more of these criteria). Both categories were retained for downstream analyses, as soft-fail variants had been detected in the initial screening performed with TB-Profiler v4.4.2, the version available at the time of isolate selection.

Identified variants were subsequently cross-referenced with the WHO mutation catalog v2 (3), with each variant assigned its corresponding category classification as defined in the WHO catalogue (File S4 to S6).

### Isolate selection

From available valid WGS data (Fig. 1), we preselected isolates carrying mutations in tier-1 genes potentially associated with resistance to BDQ, CFZ, DLM, and PA (*mmpR5*, *atpE, pepQ, Rv1979c*, *ddn, fbiA, fbiB, fbiC, fbiD,* and *fgd1*) classified as belonging to group 3 or 4 (File S4 to S6). Subsequently, isolates carrying mutations present in more than 50% of isolates within a given sub-lineage in our data set and therefore likely representing sub-lineage-defining phylogenetic SNPs rather than potential resistance variants were excluded, as were isolates carrying mutations located more than 100 base pairs upstream of the target gene.

For BDQ and CFZ, isolates carrying predicted loss-of-function mutations in *mmpS5* or *mmpL5* were excluded, since resistance mediated by efflux pumps *c*annot be assessed in a background with a non-functional pump due to epistasis. Finally, isolates not available at the Institute of Tropical Medicine (ITM, Antwerp, Belgium) for MIC testing could not be tested for MIC, mostly because only inactivated material had been shipped due to country-specific logistical and regulatory constraints related to accessibility, shipment costs, and Category A infectious substance shipping requirements.

### Isolates processing

Following culture positivity from clinical specimens, isolates underwent subculture and were stored at −80°C. For WGS, isolates were retrieved from frozen stock, sub-cultured prior to genomic DNA extraction and subsequent sequencing. Following variant analysis and isolate selection, isolates were retrieved again from the same frozen stock, subcultured, and used for MIC determination. Each selected isolate was systematically tested for the four drugs: BDQ, CFZ, DLM, and PA, hence also serving as wild-type controls for other drugs.

### MIC determination by BMD testing

MICs were determined using an in-house BMD method adapted from the EUCAST-recommended reference method (26). Testing was performed in supplemented 7H9 (7H9-S) broth comprising Middlebrook 7H9 broth (Becton Dickinson, USA) enriched with 10% OADC (oleic acid, albumin, dextrose, catalase; Becton Dickinson, USA), 0.5% glycerol, and 0.1% casitone (Becton Dickinson, USA). The drugs under evaluation were dispensed in round-bottom-shaped, non-treated 96-well polystyrene microtiter plates using an HP D300e digital dispenser (Hewlett Packard, USA). Inner wells were prefilled with 100 µL 7H9-S, while outer wells were filled with 200 µL of water to prevent plate dehydration. Drug stock solutions were prepared as per EUCAST recommendation, while serial dilutions of the stock solutions were directly dispensed in the microtiter plate. For DMSO-dissolved drugs, the solvent concentration was normalized to assure 0.2% DMSO in all wells, while for aqueous-based drugs, 5% Tween 20 was added in the stock solution to allow proper dispensing, assuring a final concentration of 0.03% Tween 20. Dispensed plates were stored at −20°C for up to 10 weeks, except for DLM, which was dispensed freshly on the day of use.

### Plate inoculation, incubation, and reading

After thawing at room temperature, the plates were shortly spun at 1,400 rpm for ±20 s. Using filter tips, 100 µL of the 10^−2^ dilution of a McF 0.5 bacterial suspension was added to each drug-containing well and the wells for the undiluted positive growth controls with and without DMSO (=GC100%). In addition, 100 µL of the 10^−4^ suspension was added to the 1/100 diluted growth control wells with and without DMSO (=GC1%). After inoculation, the lid was placed on the plates and stuck on both sides with autoclave tape. Plates were placed in a plastic box, holding an open bijou bottle with sterile distilled water and a maximum of three plates stacked. Plastic boxes were incubated at 36°C (±2°C).

Plates were read by visual inspection using an inverted mirror on days 7, 10, and 14 after incubation. If there was still insufficient growth in the GC1% on day 14, incubation was extended for a final additional reading on day 21. MIC values were determined on the first day that all GC100% wells and at least 2/3 GC1% were positive. The MIC was defined as the lowest drug concentration at which there was no visible growth. The H37Rv reference strain (BCCM/ITM-500735; CT2008-03715) was included in each run, and results of that run could only pass if the H37Rv-MIC values fell within the expected range (File S1).

As standardized WHO-recommended CCs for BMD testing in MTBC are not yet available for all drugs, MIC values were interpreted using provisional CCs derived from the literature: 0.25 mg/L for BDQ (27), 0.5 mg/L for CFZ (28), 0.125 mg/L for DLM (27), and 2.0 mg/L for PA (11).

### Structural analysis and *in silico* prediction of mutation effects

Protein sequences were retrieved from Mycobrowser and used to generate three-dimensional structural models with AlphaFold (29). Only single amino-acid substitutions were included in the structural analyses. Insertions and deletions within coding regions, as well as premature stop codons, were not modeled using AlphaFold due to the unreliability of its structure-based predictions and interpretations. Predicted structures were obtained in macromolecular crystallographic information file format and converted to protein data bank format using Gemmi to ensure compatibility with downstream analysis tools.

The impact of amino-acid substitutions on protein stability was assessed using DynaMut2, which predicts changes in folding free energy (ΔΔG), with negative values indicating destabilizing effects and positive values indicating stabilizing effects (30). However, ΔΔG values could not be calculated with DynaMut2 for variants involving multiple mutations within the same gene or across different genes. In parallel, FoldX was used to estimate mutation-induced changes in folding free energy by explicitly modeling local physical interactions such as hydrogen bonding and steric contacts, with larger positive values indicating a stronger destabilizing effect (31). FoldX calculations were restricted to variants with a single mutation per gene, as modeling was not feasible for isolates harboring mutations in multiple genes simultaneously. Predictions from both tools were interpreted quantitatively and qualitatively and used to support the structural interpretation of the mutations

Structural visualization of resistance-associated mutations was performed using UCSF ChimeraX (32) with AlphaFold per-residue confidence scores to guide structural interpretation.

### Statistical analysis

Associations between ΔΔG and MIC values were assessed using Spearman’s rank correlation coefficient (ρ). Predicted stability values were compared between fixed (≥75% allele frequency) and non-fixed (<75%) variants using the Wilcoxon rank-sum test. A two-sided *P* value < 0.05 was considered statistically significant.

## Data Availability

Individual, de-identified data including data dictionary may be shared upon reasonable request. WGS FASTQ files have been deposited in the European Nucleotide Archive under project PRJEB95959.

## SUPPLEMENTAL MATERIAL

File S1 spectrum.02005-26-s0001.xlsx Main database of the study.

File S2 spectrum.02005-26-s0002.docx Demographic and structural data.

File S3 spectrum.02005-26-s0003.pdf Association between predicted protein stability changes and MIC values.

File S4 spectrum.02005-26-s0004.tiff Distribution of delamanid and pretomanid variants across MTB sub-lineages_1.

File S5 spectrum.02005-26-s0005.tiff Distribution of delamanid and pretomanid variants across MTB sub-lineages_2.

File S6 spectrum.02005-26-s0006.tiff Distribution of bedaquiline and clofazimine variants across MTB sub-lineages.

Supplemental legends spectrum.02005-26-s0007.docx Legends for Files S1 to S6.

ASM does not own the copyrights to Supplemental Material that may be linked to, or accessed through, an article. The authors have granted ASM a non-exclusive, world-wide license to publish the Supplemental Material files. Please contact the corresponding author directly for reuse.
