# Supplementary material for: No phenotypic resistance observed for most group-3 and -4 variants in Mycobacterium tuberculosis genes related to bedaquiline, clofazimine, delamanid, and pretomanid in a Central and West African context
Source: Microbiol Spectr. 2026 Jul 28;14(9):e02005-26. doi: 10.1128/spectrum.02005-26 (PMC13532243; doi:10.1128/spectrum.02005-26)
Supplement: Supplementary file 1 [file spectrum.02005-26-s0003.pdf]

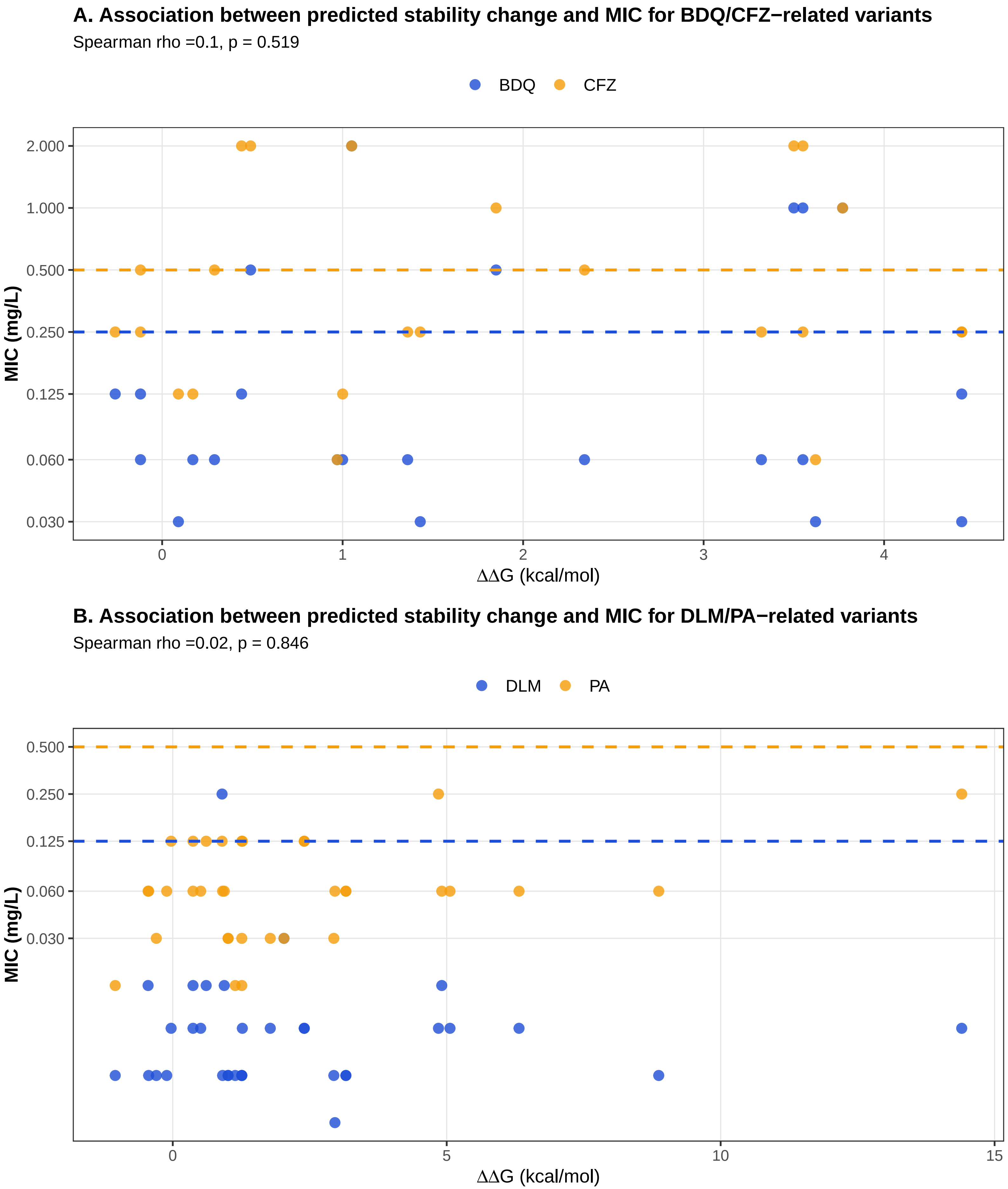
A. Association between predicted stability change and MIC for BDQ/CFZ−related variants
Spearman rho =0.1, p = 0.519
BDQ
CFZ
MIC (mg/L)
2.000
1.000
0.500
0.250
0.125
0.060
0.030
0
1
2
3
4
ΔΔG (kcal/mol)
B. Association between predicted stability change and MIC for DLM/PA−related variants
Spearman rho =0.02, p = 0.846
DLM
PA
MIC (mg/L)
0.500
0.250
0.125
0.060
0.030
0
5
10
15
ΔΔG (kcal/mol)
