# Supplementary material for: No phenotypic resistance observed for most group-3 and -4 variants in Mycobacterium tuberculosis genes related to bedaquiline, clofazimine, delamanid, and pretomanid in a Central and West African context
Source: Microbiol Spectr. 2026 Jul 28;14(9):e02005-26. doi: 10.1128/spectrum.02005-26 (PMC13532243; doi:10.1128/spectrum.02005-26)
Supplement: Supplementary file 2 [file spectrum.02005-26-s0002.docx]

Supplement 2

**Table 1 S2** : Characteristics of the samples included by lineage

|  | **L2 (n=4)** | **L3 (n=5)** | **L4 (n=72)** | **L5 (n=6)** | **L6 (n=1)** | **L8 (n=1)** | **Total (n=89)** | |
| --- | --- | --- | --- | --- | --- | --- | --- | --- |
| **Resistance to RIF** |  |  |  |  |  |  |  | |
| Yes | 3 (75.0%) | 4 (80.0%) | 36 (50.0%) | 5 (83.3%) | 1 (100.0%) | 1 (100.0%) | 50 (56.2%) | |
| No | 1 (25.0%) | 1 (20.0%) | 36 (50.0%) | 1 (16.7%) | 0 | 0 | 39 (43.8%) | |
| **Borderline rpoB mutations** |  |  |  |  |  |  |  | |
| Yes | 1 (33.3%) | 1 (25.0%) | 6 (16.7%) | 1 (20.0%) | 0 | 1 (100.0%) | 9 (18.4%) | |
| No | 2 (66.7%) | 3 (75.0%) | 30 (83.3%) | 4 (80.0%) | 1 (100.0%) | 0 | 41 (71.6%) | |
| **Retreatment case** |  |  |  |  |  |  |  | |
| Yes | 2 (50%) | 4 (75.0%) | 59 (81.9%) | 4 (66.7%) | 1 (100%) | 0 | 70 (78.7%) | |
| No | 2 (50%) | 1 (25.0%) | 13 (18.1%) | 2 (33.3%) | 0 | 1 (100.0%) | 19 (21.3%) | |
| **Country of origin** |  |  |  |  |  |  |  | |
| Cameroon | 0 | 1 (20.0%) | 32 (44.0%) | 6 (100.0%) | 0 | 0 | 39 (43.8%) | |
| DRC | 0 | 0 | 18 (25.0%) | 0 | 0 | 0 | 18 (20.2%) | |
| Ethiopia | 0 | 4 (80.0%) | 0 | 0 | 0 | 0 | 4 (4.5%) | |
| Guinea | 4 (100.0%) | 0 | 12 (16.7%) | 0 | 1 (100.0%) | 0 | 17 (19.1%) | |
| Mali | 0 | 0 | 3 (4.2%) | 0 | 0 | 0 | 3 (3.4%) | |
| Rwanda | 0 | 0 | 5 (6.9%) | 0 | 0 | 1 (100.0%) | 6 (6.7%) | |
| Senegal | 0 | 0 | 2 (2.8%) | 0 | 0 | 0 | 2 (2.2%) | |
| **Resistance to FQ** |  |  |  |  |  |  |  |  |
| Yes | 1 (25.0%) | 0 | 1 (1.4%) | 0 | 0 | 0 | 2 (2.2%) |  |
| No | 3 (75.0%) | 5 (100.0%) | 71 (98.6%) | 6 (100.0%) | 1 (100.0%) | 1 (100.0%) | 87 (97.8%) |  |
| **MIC > CC in ≥1 gene** |  |  |  |  |  |  |  |  |
| Yes | 2 (50.0%) | 2 (40.0%) | 3 (4.2%) | 1 (16.7%) | 1 (100.0%) | 0 | 9 (9.0%) |  |
| No | 2 (50.0%) | 3 (60.0%) | 69 (95.8%) | 5 (83.3%) | 0 | 1 (100.0%) | 80 (91.0%) |  |

L: Lineage; RIF : Rifampicin; FQ : Fluoroquinolones, CC : Critical concentration, MIC : Minimal inhibitory concentration

| Gene_Mutation | FoldX | Dynamut2 | Interpretation |
| --- | --- | --- | --- |
| *atpE*_Ala6Val | -0,26 | -0,46 | Neutral effect |
| *atpE*_Glu61Val | **0,97** | **-0,52** | Destabilization of stability and folding |
| *mmpR5*_Ser2Ile | 0,44 | **-0,63** | Destabilization of folding |
| *mmpR5*_Leu44Pro | **3,77** | **-0,71** | Destabilization of stability and folding |
| *mmpR5*_Arg50Trp | 0,49 | -0,07 | Neutral effect |
| *mmpR5*_Leu74Val | **3,50** | **-2,29** | Destabilization of stability and folding |
| *mmpR5*_Leu95Trp | **3,55** | **-2,40** | Destabilization of stability and folding |
| *mmpR5*_Met139Ile | **1,05** | -0,46 | Destabilization of stability |
| *mmpR5*_Met139Thr | **1,85** | **-0,88** | Destabilization of stability and folding |
| *mmpR5*_Val150Phe | -0,06 | **-1,81** | Destabilization of folding |
| *pepQ*_Ala47Gly | **1,36** | **-1,12** | Destabilization of stability and folding |
| *pepQ*_Val92Gly | **4,43** | **-1,34** | Destabilization of stability and folding |
| *pepQ*_Lys94Gln | **1,43** | -0,12 | Destabilization of stability |
| *pepQ*_Gly197Arg | **3,55** | -0,18 | Destabilization of stability |
| *pepQ*_Ala210Val | 0,17 | **-0,86** | Destabilization of folding |
| *pepQ*_Gly240Val | **3,62** | **-0,85** | Destabilization of stability and folding |
| *pepQ*_Ala313Thr | 0,09 | 0,24 | Neutral effect |
| *rv1979c*_Ile104Thr | **3,32** | **-3,38** | Destabilization of stability and folding |
| *rv1979c*_Asp124Asn | -0,12 | 0,14 | Neutral effect |
| *rv1979c*_Gly214Ser | **1,00** | -0,01 | Destabilization of stability |
| *rv1979c*_Ile238Thr | **2,34** | **-1,75** | Destabilization of stability and folding |
| *rv1979c*_Asp246Ala | 0,29 | -0,12 | Neutral effect |

**Table 2 S2:** Predicted structural impact of BDQ/CFZ–related mutations using ΔΔG (kcal/mol) calculated with FoldX and Dynamut2

FoldX < –0,5 kcal/mol indicates stabilization, –0,5 to +0,5 neutral effects, and > +0,5 destabilization, For DynaMut2, –0,5 to +0,5 indicates neutral effects, < –0,5 destabilization, and > +0,5 stabilization, Destabilizing values are shown in bold, and mutations with MIC above the critical concentration are highlighted in **red**

| Gene_Mutation | FoldX | Dynamut2 | Interpretation |
| --- | --- | --- | --- |
| *ddn*_Glu121Lys | -0,30 | -0,43 | Neutral effect |
| *fbiA*_Gln27Pro | -0,44 | 0,02 | Neutral effect |
| *fbiA*_Arg177His | **0,91** | **-0,72** | Destabilization of stability and folding |
| *fbiA*_ Ile208Val\| Ala212Gly | **3,16** | **-** | Destabilization of stability |
| *fbiA*_ Ile209Leu | **£** | **-1,59** | Destabilization of stability and folding |
| *fbiA*_ Ile223Val | **1,27** | **-1,15** | Destabilization of stability and folding |
| *fbiA*_Met255Arg | **2,03** | **-0,48** | Destabilization of stability and folding |
| *fbiA_*Asp266Glu | **-1,05** | 0,08 | Destabilization of stability |
| *fbiA*_Cys280Trp | -0,45 | **-0,68** | Destabilization of folding |
| *fbiB*_ *Ser10Ala*\|Ile14Ser\|Phe23Val | **8,87** | **-** | Destabilization of stability and folding |
| *fbiB*_Phe23Val | **4,91** | **-1,55** | Destabilization of stability and folding |
| *fbiB*_Val46Glu | **5,06** | **-1,55** | Destabilization of stability and folding |
| *fbiB*_Lys51Thr | **1,11** | **-0,79** | Destabilization of stability and folding |
| *fbiB*_Asn97Thr | **1,14** | -0,34 | Destabilization of stability |
| *fbiB*_Leu132Val | **1,78** | **-1,11** | Destabilization of stability and folding |
| *fbiB*_Asp311Asn | **1,01** | **-0,96** | Destabilization of stability and folding |
| *fbiB*_Leu447Arg | 0,12 | **0,5** | Destabilization of folding |
| *fbiC*_Pro18Leu | -0,03 | -0,33 | Neutral effect |
| *fbiC*_Ile263Val | **1,26** | **-1,4** | Destabilization of stability and folding |
| *fbiC*_Gln400Pro | **2,96** | -0,13 | Destabilization of stability |
| *fbiC*_Arg619Cys | **0,90** | **0,5** | Destabilization of stability and folding |
| *fbiC*_Met812Val | **2,40** | **-0,68** | Destabilization of stability and folding |
| *fbiD*_Gly145Arg | **2,94** | 0 | Destabilization of stability |
| *fbiD*_His213Gln | 0,37 | 0,14 | Neutral effect |
| *fgd1*_Tyr155Ser\|Tyr163Asn | **14,4** | **-** | Destabilization of stability |
| *fgd1*_Cys194Trp | **0,51** | **-1,81** | Destabilization of stability and folding |
| *fgd1*_ Lys270Met | -0,11 | **0,99** | Destabilization of folding |
| *fgd1*_Ala271Asp | 0,11 | **-1,68** | Destabilization of folding |

**Table 3 S2,** Predicted structural impact of DLM/PA-related mutations using ΔΔG (kcal/mol) calculated with FoldX and Dynamut2

FoldX < –0,5 kcal/mol indicates stabilization, -0,5 to +0,5 neutral effects, and > +0,5 destabilization, For DynaMut2, -0,5 to +0,5 indicates neutral effects, < -0,5 destabilization, and > +0,5 stabilization, Destabilizing values are shown in bold, and mutations with MIC above the critical concentration are highlighted in **red**
