# Supplementary material for: No phenotypic resistance observed for most group-3 and -4 variants in Mycobacterium tuberculosis genes related to bedaquiline, clofazimine, delamanid, and pretomanid in a Central and West African context
Source: Microbiol Spectr. 2026 Jul 28;14(9):e02005-26. doi: 10.1128/spectrum.02005-26 (PMC13532243; doi:10.1128/spectrum.02005-26)
Supplement: Supplementary file 3 [file spectrum.02005-26-s0007.docx]

**Supplementary legends**

**Supplement 1. Main database of the study.**

Excel workbook containing the main dataset used for the study and associated quality-control information. The workbook includes separate sheets describing abbreviations and legend terms, sample inclusion criteria, the main database of isolates selected for MIC testing, H37Rv quality-control MIC ranges, TB-Profiler variant quality-control criteria, variant-level quality-control information, and genomic alignment of variants with WHO catalogue-defined regions.

**Supplement 2. Demographic and structural data.**

Supplementary tables summarizing the demographic and microbiological characteristics of the included isolates by lineage, together with predicted structural impacts of BDQ/CFZ- and DLM/PA-related mutations based on ΔΔG values calculated using FoldX and DynaMut2.

**Supplement 3. Association between predicted protein stability changes and MIC values.**
Scatter plots showing the relationship between predicted changes in protein stability (ΔΔG. kcal/mol). calculated using FoldX. and minimum inhibitory concentrations (MIC. mg/L) for **(A)** BDQ/CFZ-related variants and **(B)** DLM/PA-related variants. According to FoldX. ΔΔG values < -0.5 kcal/mol indicate protein stabilization. values between −0.5 and +0.5 kcal/mol indicate neutral effects. and values > +0.5 kcal/mol indicate destabilization. Each point represents a single isolate. with colors indicating the corresponding drug. Dashed horizontal lines represent the critical concentrations used as microbiological thresholds.

**Supplement 4: Distribution of delamanid and pretomanid variants across *Mycobacterium tuberculosis* sub-lineages_1**
Heatmap showing the proportion of isolates carrying each variant within individual sub-lineages in *ddn* **(A)**. *fbiA* **(B)**. *fbiB* **(C)** and *fbiD* **(D)**. Variant positions on the X-axis are presented with either amino-acid coordinates (e.g. S2I) or coding DNA (e.g. c.707G>T) coordinates in case of synonymous mutations. The Y-axis lists the sub-lineages with their corresponding sample sizes (n). Each cell displays the percentage of isolates harboring the corresponding variant. while colors indicate the WHO catalogue classification of the variant. Variant labels in the X-axis shown in bold correspond to group 3 or 4 variants that are present in ≤50% of isolates within the corresponding sub-lineage. For promoter variants, only those located upstream of position -100 are displayed in bold. Variants marked with an asterisk (*) represent those for which MIC testing was performed and included in the study.

**Supplement 5: Distribution of delamanid and pretomanid variants across *Mycobacterium tuberculosis* sub-lineages_2**
Heatmap showing the proportion of isolates carrying each variant within individual sub-lineages in *fbiC* **(A)** and *fgd1* **(B).** Variant positions on the X-axis are presented with either amino-acid coordinates (e.g. S2I) or coding DNA (e.g. c.707G>T) coordinates in case of synonymous mutations. The Y-axis lists the sub-lineages with their corresponding sample sizes (n). Each cell displays the percentage of isolates harboring the corresponding variant. while colors indicate the WHO catalogue classification of the variant. Variant labels in the X-axis shown in bold correspond to group 3 or 4 variants that are present in ≤50% of isolates within the corresponding sub-lineage. For promoter variants, only those located upstream of position -100 are displayed in bold. Variants marked with an asterisk (*) represent those for which MIC testing was performed and included in the study.

**Supplement 6: Distribution of bedaquiline and clofazimine variants across *Mycobacterium tuberculosis* sub-lineages**
Heatmap showing the proportion of isolates carrying each variant within individual sub-lineages in *atpE* **(A)**. *mmpR5* **(B)**. *pepQ* **(C)** and *Rv1979c* **(D)**. Variant positions on the X-axis are presented with either amino-acid coordinates (e.g. S2I) or coding DNA (e.g. c.707G>T) coordinates in case of synonymous mutations. The Y-axis lists the sub-lineages with their corresponding sample sizes (n). Each cell displays the percentage of isolates harboring the corresponding variant. while colors indicate the WHO catalogue classification of the variant. Variant labels in the X-axis shown in bold correspond to group 3 or 4 variants that are present in ≤50% of isolates within the corresponding sub-lineage. For promoter variants, only those located upstream of position -100 are displayed in bold. Variants marked with an asterisk (*) represent those for which MIC testing was performed and included in the study
